# Real-world outcomes in patients with malignancy and moderate-to-severe psoriasis treated with guselkumab

**DOI:** 10.1016/j.jdin.2024.02.019

**Published:** 2024-04-07

**Authors:** Tamara Gracia Cazaña, Josep Riera Monroig, Rosa Izu, Ignacio Yanguas, Marta Lorda Espés, María Pilar Sánchez Salas, Miguel Fernando García Gil, Alba Navarro Bielsa, Beatriz Aldea Manrique, Manuel Almenara Blasco, Francisco Javier García-Latasa de Araníbar, Victoria Fuentelsaz, Ana Morales Callaghan, Mariano Ara-Martín

**Affiliations:** a Department of Dermatology, Miguel Servet University Hospital, Instituto de Investigación Sanitaria de Aragón, University of Zaragoza, Zaragoza, Spain; b Department of Dermatology, Hospital Clinic de Barcelona, Universitat de Barcelona, Barcelona, Spain; c Department of Dermatology, Basurto University Hospital, Bilbao, Spain; d Department of Dermatology, Complejo Hospitalario de Navarra, Pamplona, Spain; e Department of Dermatology, Lozano Blesa University Hospital Zaragoza, Zaragoza, Spain; f Dermatology Unit, Hospital de Barbastro, Huesca, Spain; g Dermatology Unit, Hospital Obispo Polanco, Teruel, Spain; h Dermatology Unit, San Pedro Hospital, Logroño, Spain; i Department of Dermatology, Hospital Royo Villanova, Zaragoza, Spain

**Keywords:** individualized medicine, malignancies, psoriasis

## Abstract

**Background:**

The treatment of psoriasis in patients with a personal history of cancer is a matter of debate and limited evidence is available to guide clinicians.

**Objectives:**

To report a multicenter real-life experience of a group of patients with psoriasis undergoing treatment with guselkumab and a history of cancer.

**Methods:**

We conducted a multicenter retrospective Spanish study enrolling patients with moderate-to-severe plaque psoriasis and neoplasia being treated with guselkumab for their psoriasis.

**Results:**

Twenty patients with moderate-to-severe psoriasis and at least 12 weeks of ongoing treatment were included. For the analysis, a 52 week follow-up period was evaluated in terms of efficacy and safety. Most of the malignancies in these patients were solid tumors. The percentage of patients achieving psoriasis area and severity index ≤3 at week 12 and week 52 was 80% and 87.5%, respectively, whereas 68.8% of patients achieved psoriasis area and severity index ≤1. A 52-week survival rate of 100% in the study population was observed (*n* = 20), including those patients with concomitant active cancers (*n* = 14). No adverse effects or dropouts related to guselkumab safety profile were detected.

**Limitations:**

Modest sample size and the retrospective nature of the study.

**Conclusion:**

Guselkumab not only demonstrates high effectiveness in treating psoriasis but also exhibits a favorable safety profile in patients with neoplasms.

**Capsule Summary**

• The treatment of psoriasis in patients with a personal history of cancer has limited evidence.
• This article provides evidence in real clinical practice of the use of guselkumab in patients with psoriasis and concomitant neoplasms, increasing the therapeutic arsenal.

## Introduction

Psoriasis is a chronic inflammatory skin disease that affects millions of people worldwide, posing a significant physical and psychological burden on those afflicted. It is characterized by the presence of red, scaly plaques on the skin, often accompanied by itching, pain, and social stigmatization. The complex pathogenesis of psoriasis involves the interplay of genetic, immunological, and environmental factors. Beyond the physical challenges it presents, psoriasis has also been associated with an increased risk of malignant neoplasms, adding a layer of concern for both patients and clinicians.^1^

This association between psoriasis and malignancy has sparked considerable interest in recent years, as researchers aim to unravel the complex relationship between these 2 conditions. Several factors contribute to this connection, including an underlying imbalance in immunosurveillance, chronic inflammation, long-term immunosuppressive treatments, and shared risk factors with the general population.^2^^,^^3^

One of the key players in the immune dysregulation observed in psoriasis is the interleukin 23 (IL-23)/T helper 17 pathway. IL-23 is a cytokine that plays a pivotal role in orchestrating the proinflammatory response in psoriasis. The IL-23/T helper 17 axis is involved in the recruitment and activation of immune cells, resulting in the characteristic inflammation and epidermal hyperproliferation seen in psoriatic lesions.^4^^,^^5^ Understanding the significance of IL-23 in the pathogenesis of psoriasis has paved the way for the development of targeted therapies aimed at this specific pathway.^6^^,^^7^

Guselkumab, the first IL-23 subunit p19 inhibitor human monoclonal antibody approved in Europe and the United States, has emerged as a potent therapeutic option for patients with moderate-to-severe psoriasis and psoriatic arthritis.

Patients with psoriasis and a personal history of malignancy constitute a unique population, often excluded from clinical trials due to safety concerns and the need for a more cautious approach to their care. Recognizing the dearth of evidence in this specific context, our study aims to contribute to the body of knowledge by reporting a real-life multicenter experience involving a cohort of patients with moderate-to-severe psoriasis undergoing treatment with guselkumab and with a history of cancer.^8^

## Materials and methods

A multicenter, retrospective study was designed to collect both safety and efficacy data on patients with moderate-to-severe psoriasis and neoplasia being treated with guselkumab 100 mg (weeks 0, 4 and then every 8 weeks) for their psoriasis.

The target population was patients >18 years of age who came to the dermatology clinic and were being treated with guselkumab and had a diagnosis of neoplasm in the last ≤5 years. Those with a follow-up period of <12 weeks were excluded.

Sociodemographic characteristics were collected: age and gender; variables related to psoriasis—years with psoriasis, type, psoriasis area and severity index (PASI), body surface area (BSA), dermatology life quality index (DLQI), and visual analog scale (VAS) pruritus scores; and information related to the treatment onset, induction dose, maintenance dose, and concomitant treatment. And finally variables related to their neoplasm: years since diagnosis, type of neoplasm, treatment received, active or in remission, and stage. Adverse effects (AEs) and serious AEs include events that result in death were reported.

### Statistical analysis

Descriptive statistical analysis was performed for all variables. Continuous variables were presented as the number of valid cases, mean, SD, and 25th and 75th percentiles depending on the results of the Kolmogorov-Smirnov test. Categorical variables were presented as the mean of absolute and relative frequencies of each category over the total number of valid values (*N*).

Statistical analysis was performed using STATA software for Windows 10 (Statacorp). Prior to all analyses, normality and homoscedasticity were assessed by Shapiro-Wilk and Levene tests, respectively. When data showed homoscedasticity, pairwise Student *t* test and Mann-Whitney U test were respectively used for normally and nonnormally distributed variables. When data did not showed homoscedasticity, Welch and Median tests were respectively used for normally and nonnormally distributed variables. Analysis of variance and posterior Bonferroni tests were used for multiple comparisons of normally distributed variables. Kruskal-Wallis and posterior Bonferroni-adjusted Mann-Whitney U tests were used for multiple comparisons of nonnormally distributed variables. Survival curves were compared with long-rank test.

### Ethical concerns

The present study was strictly observational, and the protocol was approved by the Aragón Ethical Committee for Clinical Research (C.I. PI23/031). All participants provided written informed consent prior to their enrolment.

## Results

### Baseline demographics and disease characteristics

**Fig 1:**
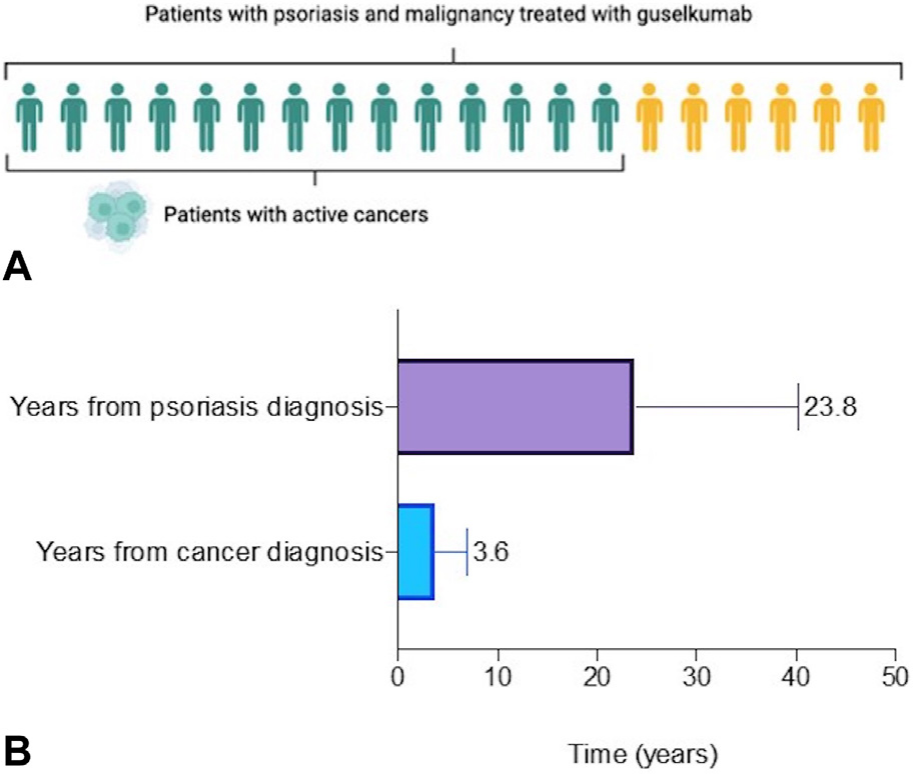
**A,** Graphical abstract of the treated population. In green 14 patients with moderate-to-severe psoriasis and active cancers treated with guselkumab. In yellow 6 patients with moderate-to-severe psoriasis and nonactive cancers treated with guselkumab. **B,** Average time (in years) from psoriasis diagnosis and cancer diagnosis of the treated population using guselkumab (*n* = 20).

For this real-world analysis 20 patients with moderate-to-severe psoriasis and at least 12 weeks of ongoing treatment were included. All patients were treated with guselkumab according to the approved label, with 14 out of 20 patients having active cancers (Fig 1, *A*). A mean of 24.3 years elapsed since psoriasis diagnosis, and the mean time from cancer diagnosis was 3.1 years (Fig 1, *B*).

For the analysis, a 52 week follow-up period was evaluated in terms of efficacy and safety. The mean follow-up time in the cohort was 36 weeks, and the maximum and median follow-up period was 52 weeks.

**Fig 2:**
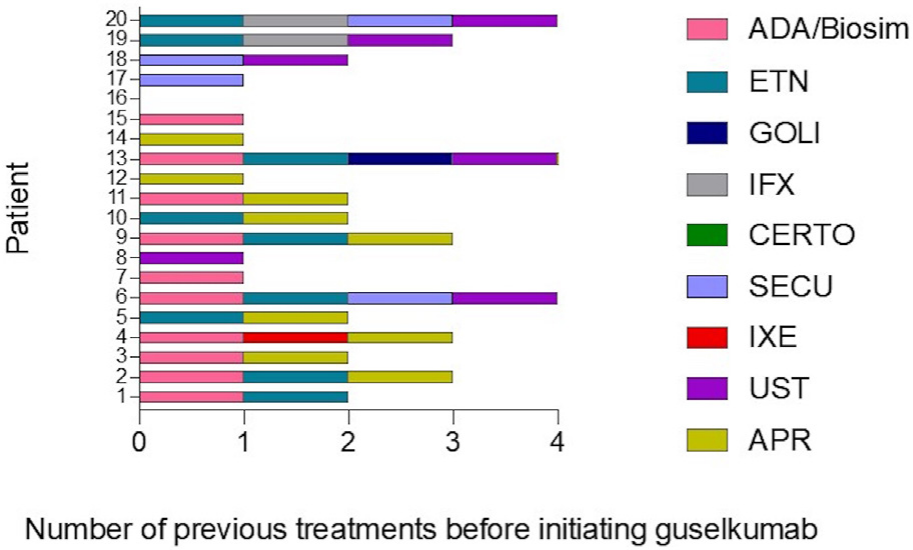
Detailed cancer classification of the treated population. X-axis indicates the number of patients treated with guselkumab with a diagnosis of the specified cancer in y-axis. *ADA*, Adalimumab; *APR*, apremilast; *Certo*, certolizumab; *ETN*, etanercept; *Goli*, golimumab; *IFX*, infliximab; *IXE*, ixekizumab; *Secu*, secukinumab; *UST*, ustekinumab.

All patients were previously treated with systemic and biologic therapies being adalimumab (*n* = 10), etanercept (*n* = 9), and apremilast (*n* = 9), the most frequent drugs used in these patients. The mean number of biologic therapies prior to guselkumab in the treated population was 2.15. Prior to the switch to guselkumab, the most frequent previous treatment was apremilast (*n* = 10), followed by ustekinumab (*n* = 6) (Fig 2).

**Fig 3:**
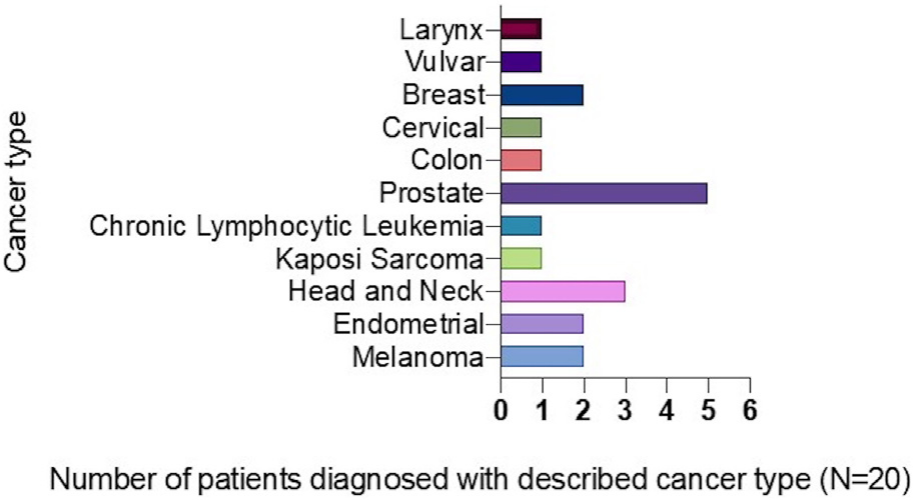
Cancer classification of the treated population. X-axis indicates the number of patients treated with guselkumab with a diagnosis of the specified cancer in y-axis.

Most of the malignancies in these patients were solid tumors, being head and neck cancer (*n* = 3) melanoma (*n* = 2), and endometrial cancer (*n* = 2) the most frequent neoplasms. Vulvar (*n* = 1), breast (*n* = 1), cervical (*n* = 1), colon (*n* = 1), prostate (*n* = 1), chronic lymphocytic leukemia (*n* = 1), Kaposi sarcoma (*n* = 1), and larynx (*n* = 1) cancers were other tumor types present in these cohort of patients (Fig 3).

### Clinical outcomes

**Fig 4:**
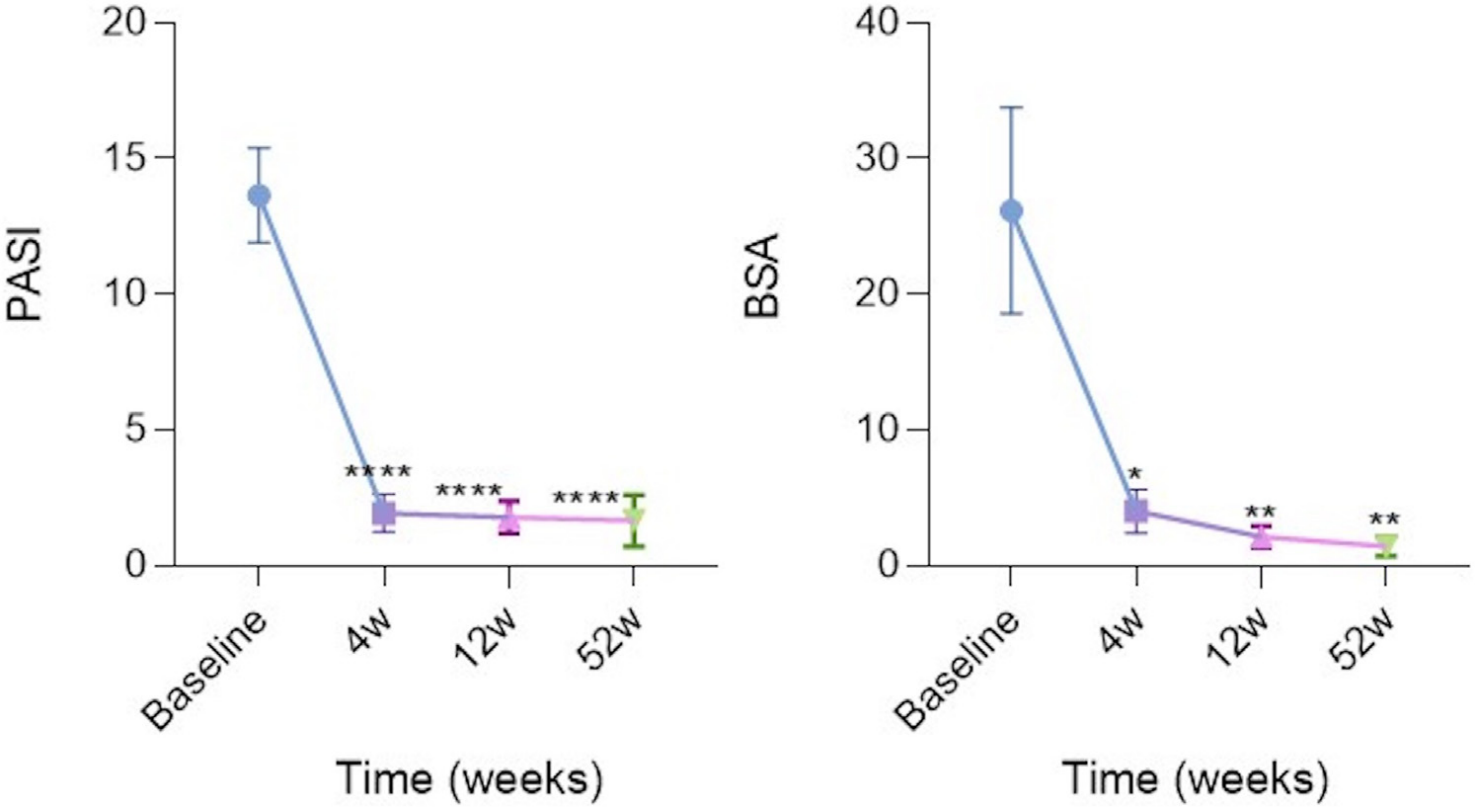
Absolute psoriasis area and severity index (PASI; *left*) and body surface area (BSA; *right*) score from baseline to week 4, week 12, and week 52 in the overall population (*n* = 20). Error bars correspond to SD. *P* values obtained using Student *t* test. ∗*P* < .05; ∗∗*P* < .01; ∗∗∗∗*P* < .0001.

Regardless of previous psoriasis treatment history, absolute PASI score was significantly reduced from baseline to week 4, showing high response rates and fast onset of action in all patients. Between weeks 4 and 12 absolute PASI continued to decrease showing the greatest reduction at week 52. A reduction of 12 points in absolute PASI from baseline to week 12 was observed. The mean absolute PASI values were 13.6 ± 7 for baseline, 1.9 ± 2.05 for week 4, 1.8 ± 2.3 for week 12, and 1.65 ± 3.8 for week 52. The percentage of patients achieving PASI ≤3 at week 12 and week 52 was 80% and 87.5%, respectively, whereas 68.8% of patients achieved PASI ≤1 (Fig 4, *left*).

BSA values were also collected and analyzed. BSA was significantly reduced from baseline to week 4 displaying the greatest reduction at week 52. The mean BSA values were 26.1 ± 30.35 for baseline, 4 ± 4.54 for week 4, 2.10 ± 3 for week 12, and 1.4 ± 2.6 for week 52 (Fig 4, *right*).

Patient-reported outcomes were also analyzed in these patients. Pruritus VAS and DLQI score were performed, evaluating the efficacy of the treatment from baseline to week 52. The DLQI score decreased from a mean of 9.3 at baseline to 1.7 at 4 weeks, 1.4 at 12 weeks, and 0.45 at week 52. Similar improvements were seen in pruritus VAS: from a mean baseline value of 4.4, decreasing to 1.6 at 4 weeks, to 0.6 at 12 weeks, and to 0.00 at 52 weeks.

**Fig 5:**
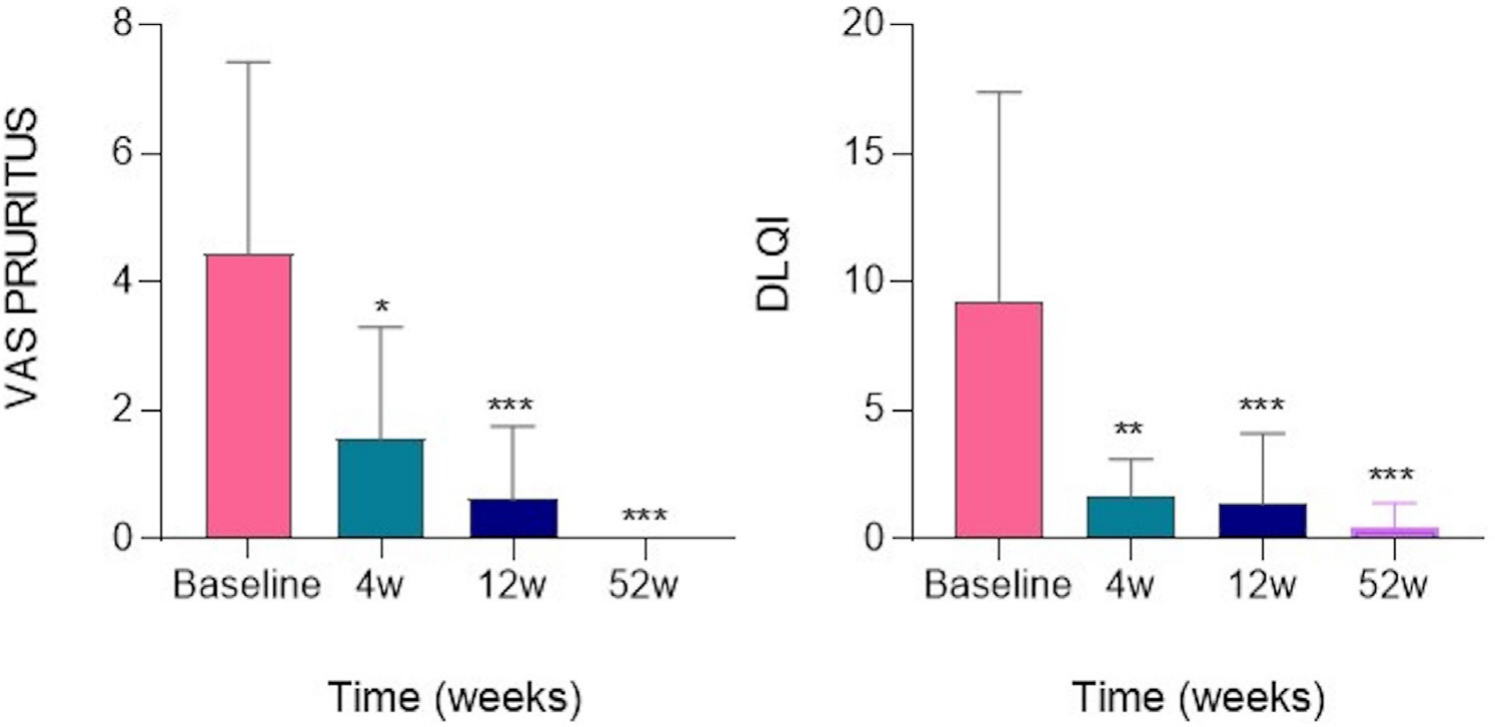
Dermatology life quality index (DLQI) and pruritus visual analog scale (VAS) score from baseline to week 4, week 12, and week 52 in the overall population (*n* = 20). The number of each group indicates the mean pruritus VAS score (*left graph*) and DLQI score (*right graph*). Error bars correspond to SD. *P* values obtained using Student *t* test. ∗*P* < .05; ∗∗*P* < .01; ∗∗∗*P* < .001.

The mean DLQI score values were 9.3 ± 8.1 for baseline, 1.7 ± 1.4 for week 4, 1.4 ± 2.7 for week 12, and 0.45 ± 0.9 for week 52. The mean pruritus VAS score values were 4.4 ± 3 for baseline, 1.6 ± 1.7 for week 4, 0.6 ± 1.1 for week 12, and 0.0 ± 0.0 for week 52 (Fig 5).

### Drug survival and safety

A 52-week survival rate of 100% in the study population was observed (*n* = 20), including those patients with concomitant active cancers (*n* = 14). No AEs, neither serious AEs nor dropouts related to guselkumab safety profile, were detected in the nonactive cancer group or in the active-cancer group of patients.

## Discussion

In our study, we report the real-life experience of 20 patients with moderate-to-severe psoriasis who were treated with guselkumab and had newly diagnosed neoplasia. Notably, no tumor recurrence or progression was observed during the initial 52 weeks of treatment. Furthermore, our data confirmed the high effectiveness of guselkumab, with statistically significant reductions in PASI, DLQI, BSA, and VAS pruritus scores after 52 weeks of treatment.

Chronic inflammation has long been associated with an increased risk of malignancy. IL-23 serves as a crucial molecular link between tumor-promoting proinflammatory processes and the failure of adaptive immune surveillance to infiltrate tumors. Expression of IL-23 in human tumors, which can modulate local inflammatory responses within the tumor microenvironment and influence the infiltration of intraepithelial lymphocytes. Genetic deletion or antibody-mediated elimination of IL-23 enhances the infiltration of cytotoxic T cells into transformed tissue, offering protective effects against carcinogenesis.^9^

Guselkumab’s effectiveness extends to addressing the full spectrum of psoriatic manifestations, offering rapid and sustained responses with a commendable safety profile.^10^^,^^11^ Guselkumab has been also associated with a limited number of malignancy cases in preclinical trials and the overall rates of malignancy in a larger cohort of guselkumab-treated patients were consistent with those expected in the general population.^12^

In this context, it is crucial to acknowledge that the available evidence is, to date, limited. Clinical trials, including the VOYAGE 1 and VOYAGE 2 trials, have reported only a small number of malignancy cases among guselkumab-treated patients. These cases included prostate cancer, breast cancer, and nonmelanoma skin cancers.^10^ However, the overall rates of malignancy over extended periods of follow-up have been reassuringly low and consistent with expected rates in both the general population and a real-world cohort of psoriasis patients.^11^^,^^12^

Amidst these advancements, the treatment of psoriasis in patients with a personal history of cancer remains a challenging area of clinical decision-making. There is a paucity of robust evidence to guide health care professionals in these scenarios, necessitating careful and individualized consideration of treatment options.^13^ The importance of shared decision-making between dermatologists and oncologists cannot be overstated, as it is crucial to weigh the potential benefits of psoriasis treatment against the risk of cancer recurrence.^8^

Together with the reported data in this article, growing evidence about the efficacy and safety of guselkumab in cancer treated patients is emerging.^14^ Kamiya et al^15^ reported successful psoriasis vulgaris treatment with guselkumab in a patient with non–small cell lung cancer, and Mastorino et al^8^ reported 7 patients with a history of cancer and psoriasis who received guselkumab, with no data indicating neoplasm recurrence at 12 months of follow-up.

In light of this evidence, it can be concluded that inhibiting IL-23 with guselkumab not only offers a high response efficacy in psoriasis but also maintains a good safety profile in patients with neoplasms.^6^^,^^7^^,^^11^^,^^16^ However, it is essential to acknowledge the limitations of our study, which may affect the generalizability of the results. These limitations include the relatively small number of patients, heterogeneity in cancer types, the inclusion of early-stage tumors, and the relatively short duration of observation.

It is imperative to foster collaboration between dermatologists, oncologists, and researchers to conduct rigorous studies that address the specific concerns of psoriasis patients with a history of cancer. These studies should aim to establish standardized treatment protocols that optimize both the efficacy and safety of treatment, ultimately offering patients the best possible care. As we advance in our understanding of the connections between psoriasis, cancer, and novel therapies, we strive to provide patients with the most effective and evidence-based treatments while ensuring their overall well-being and quality of life.

In conclusion, the current evidence suggests that there is no direct association between anti-IL-23 agents, such as guselkumab, and the development of cancer. Given the absence of cancer recurrence in other case series and the promising results of our study, there is no evidence to exclude guselkumab in psoriatic patients with a prior cancer diagnosis.

## Conflicts of interest

Dr Gracia Cazaña has received grants for attending congresses as well as fees for studies, lectures, presentations, courses, and consultancy from AbbVie, Almirall, Amgen, Biogen, Janssen, Lilly, Leo, Novartis, Pfizer, and UCB. Dr Yanguas has received grants for attending congresses as well as fees for studies, lectures, presentations, courses, and consultancy from AbbVie, Leo Pharma, Novartis, Janssen, Lilly, Amgen, Almirall, Pfizer, and MSD. Drs Morales Callaghan and Ara-Martín have received grants for attending congresses as well as fees for studies, lectures, presentations, courses, and consultancy from Janssen, Novartis, Lilly, AbbVie, Leo Pharma, Almirall, UCB Pharma, Sanofi, and Pfizer. Drs Riera Monroig, Izu, Lorda Espés, Sánchez Salas, García Gil, Navarro Bielsa, Aldea Manrique, Almenara Blasco, García-Latasa de Araníbar, and Fuentelsaz have no conflicts of interest to declare.
